# Traditional Chinese Medicine-Guided Dietary Intervention for Male Youth Undergoing Drug Detoxification: A Randomized Controlled Trial

**DOI:** 10.1155/2019/3870316

**Published:** 2019-12-03

**Authors:** Li-wan Zhang, Qing Guo, Rui Fang, Li Lin, Bin-hua Ye, Kai-lin Zheng, Min Lin, Zhao-yang Yang, Ji-qian Fang, Can-dong Li

**Affiliations:** ^1^School of Medicine, Hangzhou Normal University, Hangzhou, China; ^2^People's Hospital Affiliated to Fujian University of Traditional Chinese Medicine, Fuzhou, China; ^3^Juvenile Compulsory Isolated Detoxification Center of Fujian Province, Fuzhou, China; ^4^Fujian University of Traditional Chinese Medicine, Fuzhou, China; ^5^School of Public Health, Sun Yat-Sen University, Guangzhou, China

## Abstract

**Objective:**

The aim of this study was to evaluate the effectiveness of traditional Chinese medicine- (TCM-) guided dietary interventions in improving yang-qi deficiency and yin-blood deficiency TCM syndromes according to the principles of TCM syndrome differentiation theory in male youths undergoing drug detoxification during the rehabilitation period who stayed in a compulsory isolation detoxification center.

**Methods:**

Male youths undergoing drug detoxification who met the criteria to be included in the study were randomly divided into the intervention group (*n* = 62) and the control group (*n* = 61) according to a random number table in a 1 : 1 ratio. The intervention group received a TCM-guided diet, and the control group received routine food support. Over an intervention period of 3 months, we observed changes in the TCM syndrome element scores in the two groups before and after intervention.

**Results:**

After 3 months, the qi deficiency, yin deficiency, blood deficiency, and yin-blood deficiency syndrome in the intervention group improved significantly (*P* values 0.009, 0.000, 0.005, and 0.001, respectively). In the control group, yang deficiency, qi deficiency, and yang-qi deficiency syndromes worsened significantly (*P* values 0.003, 0.032, and 0.009, respectively). The differences (post-pre) in yang deficiency, qi deficiency, yang-qi deficiency, yin deficiency, blood deficiency, and yin-blood deficiency syndromes between the two groups were statistically significant (*P* values 0.003, 0.003, 0.003, 0.001, 0.005, and 0.002, respectively).

**Conclusion:**

A TCM-guided diet can delay the worsening of yang-qi deficiency syndrome symptoms and improve yin-blood deficiency syndrome and the prognosis of male youth undergoing drug detoxification during the rehabilitation period.

## 1. Introduction

Drug addiction has become a common social topic worldwide and a serious public health problem, causing very large economic and social burdens worldwide. Addictive drugs are also known as psychoactive substances, including alcohol, nicotine, opioids, marijuana, cocaine, amphetamine-type stimulants, and hallucinogens. Among these drugs, heroin, marijuana, hallucinogens, cocaine, etc., which are prohibited by law, are called illegal drugs (illegal/illicit drugs) [1]. The 2016 World Drug Report by the United Nations showed that approximately 2.5 million people had used at least one drug [2], equivalent to 5% of the adult population, and 207 thousand people died after using the drugs in 2014 [3]. After nearly ten years, mortality associated with heroin and cocaine use has increased by 5 times compared with the rates in 2000 in the USA [4, 5]. According to a new investigation in China, from 2003 to 2010, the proportion of heroin abusers among new drug users dropped by 52.3% [6], but there were 2.505 million drug users in China, of which there were still 955 thousand opiate abusers in 2016 [7]. In 2012, the Drug Abuse Monitoring Annual Report of China showed that the key populations for targeting drug abuse prevention are the youth aged 25 and below, accounting for 48.2% of the total number of people with drug addictions. The juvenile drug abusers mainly took amphetamine-type synthetic drugs with excitatory and hallucinogenic effects, and new synthetic drug abusers accounted for 81% of new drug abusers [7]. The detoxification of juvenile drug abusers still requires a great deal of progress, and the abuse of new synthetic drugs is also spreading rapidly in China.

Drug addiction is a type of chronic recurrent encephalopathy. Due to long-term repeated use of addictive substances such as heroin, marijuana, and morphine, patients have a strong physical and mental dependence, which leads to persistent cravings for drugs, compulsive use of drugs, and relapse behaviour after withdrawal [8]. Excessive use of addictive drugs will not only cause acute poisoning in the body but can also cause serious pathological damage to the central nervous system, cardiovascular system, respiratory system, and urinary system and even death [9–12]. The relapse of drug users after detoxification is not only a difficult challenge in drug treatment but is also a problem in terms of global drug control and drug rehabilitation strategies. Studies have shown that the relapse rate is above 80% in Western countries with advanced detoxification conditions and methods, while the relapse rate is generally above 90% in China [13]. The prevention of relapse is the key to detoxification in individuals with addictive behaviours [14].

Currently, interventions for relapse prevention in patients with drug addiction mainly include drug detoxification treatment and psychological behaviour interventions. Drug detoxification treatment includes alternative medicine treatments and nonalternative medicine treatments, mainly focusing on the physical dependence of the addicted individual and on reducing the withdrawal symptoms of the patient [15, 16]. The aetiology of drug addiction is very complicated, which involves multiple brain regions, receptor systems, and various intracellular signal pathways [17]. Since the mechanism is not yet well understood, the ideal treatment for addicts' relapse remains unknown either.

Since the late 19th century, Chinese medical practitioners began to study opium addiction [18]. Traditional Chinese medicine (TCM) plays a unique role in drug detoxification. With treatment based on syndrome differentiation, relatively small adverse reactions, and obvious clinical effects, it has drawn attention and provided new ideas for research on drug detoxification [19]. In TCM, there is no such theory called “addictive memory,” and most practitioners call it “opium addiction,” “drug cessation syndrome,” “drug addiction,” “heart addiction,” and so on. Although Chinese medicine is not very clear about the aetiology or pathogenesis of benzedrine-based drugs that induce addiction, the opinion on the main pathogenic process that results in drug addiction in the context of TCM is relatively consistent: the long-term excessive use of drugs accumulates the drug poison in the viscera and generates phlegm, stasis of blood, poisonous elements, and other pathological products. Long-term drug use affects the functions of the internal organs, which, over time, leads to the loss of qi, blood, and body fluids and the imbalance of yin and yang. Therefore, drug addiction is often accompanied by characteristic deficiency syndrome.

According to the *Guidelines on Dietary Nutrition for Drug Detoxification Personnel under Forced Isolation* [20], scientific and reasonable provision of dietary nutrition, formulation of diets for drug abusers, and reasonable adjustment of diets can help drug abusers reduce withdrawal reactions, promote physical rehabilitation, and maintain safety and stability. The TCM culture has a long history, and drug detoxification using TCM has occurred for more than 200 years with unique therapeutic advantages. As the essence of Chinese medical culture, TCM-guided diets feature the integration of medicine and food and the combination of recuperation and treatment and embodies the thought of treatment based on syndrome differentiation of TCM, regulating the yin-yang disharmony of people with drug addiction through diet [21–23]. Based on the syndrome element differentiation theory, this study was a randomized controlled trial on male youth abusers of amphetamine-type drugs who were treated by a TCM-guided diet to explore the clinical value of a TCM-guided diet in improving TCM syndromes of patients abstaining from drugs.

## 2. Methods

### 2.1. Study Participants and Randomization

**Figure 1:**
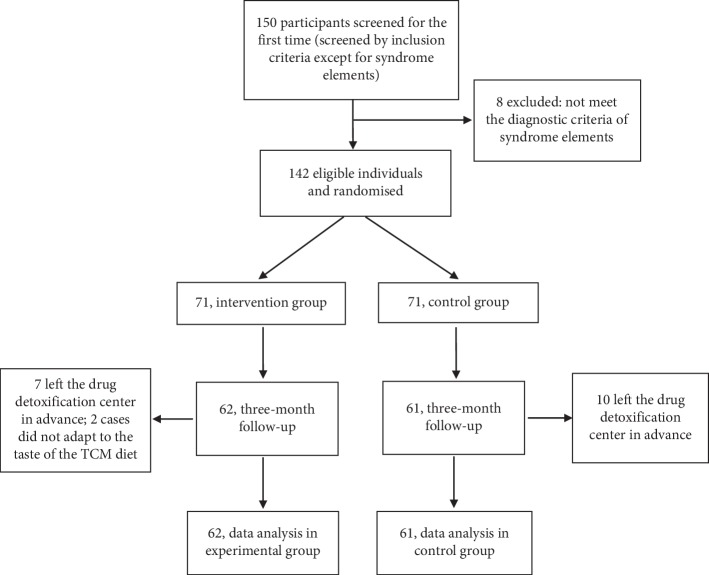
Flow of participants through the trial.

A total of 150 male youths who were forced to abstain from drugs in the Fujian Juvenile Judicial Forced Isolation Drug Detoxification Center from November 2017 to February 2019 were considered for inclusion in the study. A total of 142 male youths undergoing drug detoxification met the inclusion criteria and were randomly divided into the intervention group (*n* = 71) and the control group (*n* = 71) by using a random number table at a 1 : 1 ratio. Nineteen participants were lost to follow-up and not observed with a loss rate of 13.4%. The causes of loss or termination included that the participants met the criteria for discharge in advance (*n* = 17) or they were not adapted to the taste of the TCM-guided diet (*n* = 2). A total of 123 participants were included in the 3-month follow-up, including 62 in the intervention group and 61 in the control group. The average age in the intervention group was 22.10 ± 2.63 years old and that in the control group was 21.88 ± 2.90 years old. Recruitment and participation in the trial are described in Figure 1.

### 2.2. Diagnostic Criteria

① Drug addiction: drug abuse was defined by the World Health Organization as nonmedical use of drugs with long-term repetitive, compulsive self-medication behaviour characterized by increasing doses [1]. ② Amphetamine-type stimulants: a general term for central agglutinants converted from all amphetamines, including excitatory amphetamines, hallucinogenic amphetamines, appetite-suppressing amphetamines, and mixed amphetamines. ③ TCM syndrome differentiation: the *TCM Syndrome Scale for Drug Addicts*, developed by the TCM Syndrome Research Base of the Fujian University of Traditional Chinese Medicine, was used to determine the TCM syndrome elements of the participants, and its reliability and validity were strictly evaluated. The data were imported into the four TCM diagnostic information collection systems and syndrome differentiation systems to obtain the corresponding syndrome element scores. According to the four main syndrome elements of yang deficiency, qi deficiency, yin deficiency, and blood deficiency, if the score is more than 100 points, it is determined as a positive disease element, and for those with miscellaneous syndrome elements, the syndrome element with the highest score is determined as the principal syndrome.

### 2.3. Inclusion and Exclusion Criteria

① Patients in forced isolation who abstained from drugs; ② male youth aged 13–25; ③ those who would continue staying in the rehabilitation center for more than 3 months; ④ those with a TCM syndrome element differentiation defined as yang-qi deficiency syndrome or yin-blood deficiency syndrome; and⑤ those who agreed to sign the informed consent form for this study were included. ⑥ Participants with other diseases or comorbidities, such as diabetes, primary hypertension, or other serious primary diseases were excluded; ⑦ those with cancer, infections, active inflammation, and immune system and haematopoietic system diseases were excluded; ⑧ those who were allergic or intolerant to the nutritional diet were excluded; and ⑨ those with unconsciousness, dementia, or psychosis or people who were unable to cooperate with the study were excluded.

### 2.4. Interventions

The intervention was conducted in two phases, and each phase lasted for three months.

#### 2.4.1. Intervention Group

Patients with yang-qi deficiency syndrome were provided a TCM-guided diet that warms yang and invigorates qi; one staple food or side dish was replaced with a TCM-guided dietary component every day. The intervention group with yin-blood deficiency syndrome was provided a TCM-guided diet that nourished yin and blood, with one staple food or side dish replaced with a TCM-guided dietary component every day. To fully assess the advantages of the prescriptions, to take into account the balance of nutrition, and improve participants' compliance, 42 medicinal meals were screened, which were circulated every 21 days; the meals included 21 dishes in the warms-yang-and-invigorates-qi diet and 21 dishes in the nourishes-yin-and-blood diet, and the menus of TCM-guided diets are described in Table 1.

TCM-guided diet prescription was performed with reference to the diet of TCM [24], the medicinal diet of TCM [25], the practical nutrition of TCM [26], and other classic TCM works. The main therapeutic effects of diets prescribed for the yang-qi deficiency syndrome group were yang and qi nourishing, spleen invigorating, liver and kidney nourishing, qi and blood nourishing, etc. The main therapeutic effects of diets prescribed for the yin-blood deficiency syndrome group were yin and blood nourishing, spleen and qi invigorating, liver and kidney nourishing, etc. The selected TCM-guided diet should ensure the homology of medicine and food with no toxic or side effects, and food materials should be selected to ensure availability and easy preparation. In this study, Chinese herbs were sourced from Fuzhou Huichun Pharmaceutical Co. Ltd. The TCM-guided diet was prepared by trained cooks as required. The other food materials were sourced by the cafeteria of the Juvenile Judicial Forced Isolation Drug Detoxification Center of Fujian Province in accordance with the bidding standard.

#### 2.4.2. Control Group

The control group received a routine support diet in the drug detoxification center.

### 2.5. Study Design and Blinding

We performed a pilot single-blinded, two-group, randomized controlled trial. The allocation procedure ensured that participants, outcomes assessors, data collectors, and statisticians were blinded to the intervention throughout the trial.

### 2.6. Outcome Measurements

#### 2.6.1. Evaluation Indicators

The TCM syndrome elements were determined by the TCM Syndrome Scale for Drug Addicts. The four diagnostic methods of TCM were introduced, and the corresponding syndrome element integrals were obtained in the information collection and syndrome differentiation systems. We evaluated the improvement in TCM health status according to the integral changes of four main pathogenic syndromes: yang deficiency, qi deficiency, yin deficiency, and blood deficiency. The specific diagnostic criteria of the syndrome elements [27] are shown in Table 2.

#### 2.6.2. General Observation Indicators

The vital signs of participants, height, weight, blood pressure, and pulse were observed.

### 2.7. Sample Size

According to the literature report and preliminary preexperimental results, it was assumed that the statistically significant difference (mean) of the TCM syndrome element improvement between the two groups was 84, and the standard deviation was 120. The incidence probability of type I error was 5%, and the statistical power was 80%. The proportion of the two groups was 1 : 1. According to the formula *n* = ((2*σ*^2^(*t*_*α*_ + *t*_*β*_)^2^)/*δ*^2^), a total of 110 participants were needed, with 55 participants in each group. With an estimated loss to follow-up rate of 15%, we planned to enrol 128 participants in the two groups, with 64 participants in each group.

### 2.8. Statistical Analysis

SPSS statistical software 21.0 was used for statistical analysis. The normally distributed measurement data were all expressed as the mean ± standard deviation, while the nonnormally distributed data were expressed as the median (interquartile range) and the counting data were expressed as the frequency. For the measurement data among groups, the syndrome element scores and differences (*d*) were compared before and after treatment. Those with normal distribution and variance homogeneity underwent grouped *t*-test; otherwise, the Mann–Whitney *U* test was conducted. For the intragroup measurement data, the syndrome element scores were compared before and after treatment. Those with a normal distribution underwent the paired *t*-test; otherwise, the Mann–Whitney *U* test was used. The counting data were compared by *χ*^2^ test, and *P* < 0.05 indicated that the difference was statistically significant.

## 3. Results

### 3.1. Demographic and Health Characteristics

There were no significant differences in the demographic characteristics (age, nationality, occupation, marital status, educational level, family economic status, and medical form) between the two groups (Table 3). There were no significant differences in the drug abuse characteristics (years of drug abuse, types of drugs, drug abuse patterns, daily drug abuse, and frequency of drug detoxification) between the two groups by *χ*^2^ or *t*-test. *P* values were greater than 0.05 (Table 4).

### 3.2. Score of the Yang Deficiency Syndrome Element

There was no statistically significant difference between the intervention group and the control group in terms of the score of the yang deficiency syndrome element before the intervention (*P*=0.189 > 0.05). After the intervention with the TCM-guided diet, through intergroup comparison, it was found that the change (improvement) in the score of the yang deficiency syndrome element in the intervention group was not statistically significant (*P*=0.359 > 0.05). The change (worsening) in the score of the yang deficiency syndrome element of the rehabilitation personnel in the control group was statistically significant (*P*=0.003 < 0.05). Through intergroup comparison, the changes (post-pre) in the score of the yang deficiency syndrome element were significantly different (*P*=0.003 < 0.05) between the two groups (Table 5).

### 3.3. Score of the Qi Deficiency Syndrome Element

There was no statistically significant difference between the intervention group and the control group in terms of the score of the qi deficiency syndrome element before the intervention (*P*=0.052 > 0.05). After the intervention with the TCM-guided diet, through intergroup comparison, it was found that the change (improvement) in the score of the qi deficiency syndrome element in the intervention group was statistically significant (*P*=0.009 < 0.05), and the change (worsening) in the score of the qi deficiency syndrome element of the rehabilitation personnel in the control group was statistically significant (*P*=0.032 < 0.05). Through intergroup comparison, it was found that the changes (post-pre) in the score of the qi deficiency syndrome element were significantly different (*P*=0.003 < 0.05) between the two groups (Table 6).

### 3.4. Sum of Scores of Yang-Qi Deficiency Syndrome Elements

There was no statistically significant difference between the intervention group and the control group in terms of the sum of the scores of the yang-qi deficiency syndrome element before the intervention (*P*=0.101 > 0.05). After the intervention with the TCM-guided diet, through intergroup comparison, it was found that the change (improvement) in the sum of the scores of the yang-qi deficiency syndrome elements in the intervention group was not statistically significant (*P*=0.079 > 0.05). The change (worsening) in the sum of the scores of the yang-qi deficiency syndrome elements of the rehabilitation personnel in the control group was statistically significant (*P*=0.009 < 0.05). Through intergroup comparison, the changes (post-pre) in the sum of the scores of the yang-qi deficiency syndrome elements were significantly different (*P*=0.003 < 0.05) between the two groups (Table 7).

### 3.5. Score of the Yin Deficiency Syndrome Element

There was no statistically significant difference between the intervention group and the control group in terms of the yin deficiency syndrome element score before the intervention (*P* = 0.006 > 0.05). After the intervention with the TCM-guided diet, through intergroup comparison, it was found that the change (improvement) in the score of the yin deficiency syndrome element in the intervention group was statistically significant (*P* < 0.001 < 0.05). The change (worsening) in the yin deficiency syndrome element score of the rehabilitation personnel in the control group was not statistically significant (*P* = 0.133 > 0.05). Through intergroup comparison, the changes (post-pre) in the yin deficiency syndrome element score were significantly different (*P* = 0.001 < 0.05) between the two groups (Table 8).

### 3.6. Score of the Blood Deficiency Syndrome Element

There was no statistically significant difference between the intervention group and the control group in terms of the score of the blood deficiency syndrome element before the intervention (*P*=0.132 > 0.05). After the intervention with the TCM-guided diet, through intergroup comparison and intragroup comparison, it was found that the change (improvement) in the score of the blood deficiency syndrome element in the intervention group was statistically significant (*P*=0.005 < 0.05). The change (worsening) in the score of the blood deficiency syndrome element of the rehabilitation personnel in the control group was not statistically significant (*P*=0.065 > 0.05). Through intergroup comparison, the changes (post-pre) in the score of the blood deficiency syndrome element were significantly different (*P*=0.005) between the two groups (Table 9).

### 3.7. Sum of Scores of the Yin-Blood Deficiency Syndrome Element

There was no statistically significant difference between the intervention group and the control group in terms of the sum of the scores of the yin-blood deficiency syndrome element before the intervention (*P* = 0.088 > 0.05). After the intervention with the TCM-guided diet, through intergroup comparison, it was found that the change (improvement) in the sum of the scores of the yin-blood deficiency syndrome elements in the intervention group was statistically significant (*P* = 0.001 < 0.05). The change (worsening) in the sum of the scores of the yin-blood deficiency syndrome elements of the rehabilitation personnel in the control group was not statistically significant (*P* = 0.090 > 0.05). Based on the intergroup comparison, the changes (post-pre) in the sum of the yin-blood deficiency syndrome element scores were significantly different (*P* = 0.002 < 0.05) between the two groups (Table 10).

### 3.8. Vital Signs of the Two Groups before and after the Intervention

There were no statistically significant differences between the intervention group and the control group in height, weight, blood pressure, or pulse at baseline and after 3 months (Table 11).

## 4. Discussion

At present, because the target of chemical drug detoxification is relatively focused, the side effects are many, most drugs cause new dependence, and the problem cannot be solved fundamentally [28]. In the long-term clinical practice of drug rehabilitation, a complete scientific theory has been gradually formed on the basis of TCM. The therapeutic advantages of the TCM multisystem, multilink, and multitarget approach are playing an increasingly important role [29]. These dietary therapies rely specifically on the nutritional components of foods [30]. In the theory of TCM, the therapeutic effects of foods take into consideration the nature, tastes, and functions of the foods and their effects on human constitutions in terms of yin-yang harmony [31].

In accordance with the holistic view of the basic theory of TCM, Chinese medical practitioners using TCM-guided diets regard the human body as an organic whole centring on the five viscera with the six hollow organs, five body constituents (tendon vessel, muscle, hair, skin, and bone), five sense organs, nine orifices, and limbs and bones being connected by meridians. Since the functional activities of the human body are governed by the functions of the essential essence, qi, blood, and body fluids, the viscera and organs of the human body are inseparable in structure, are coordinated in function and mutual restriction, reinforce each other, and are mutually influential to each other in pathological development [32]. With the great importance attached to the concept of organic wholeness, a TCM-guided diet can be used for drug detoxification. The TCM-guided diet can eliminate protracted syndrome and assist the comprehensive recuperation during rehabilitation. Most TCM-guided diet therapies for drug dependence can reach such efficacies as tonifying qi, tranquillising the mind, calming patients, relieving dizziness, and stopping spasm and pain. Diet therapies can reach the whole body through digestion and absorption, unclog the body veins, regulate vegetative nerve function, enhance blood flow, activate the cells of the whole body, accelerate metabolism, and eliminate toxic substances and hepatorenal toxins in the body. The TCM-guided diet is an effective alternative therapy to assist with detoxification. In the treatment of postaddiction disorders, Chinese medical practitioners apply TCM-guided diets not only to strengthen the resistance of the body, dispel pathogenic factors, and comprehensively regulate overall body functions but also to address the main syndrome to calm the mind, relieve pain, and harmonize the intestines and stomach to achieve a key breakthrough in drug detoxification.

Based on the TCM syndrome element differentiation theory, yang-qi deficiency syndrome caused by drug addiction is mostly related to “renal function,” and the yin and yang of the kidney are the fundamental elements of yin and yang in the viscera. Therefore, the kidney is regarded by Chinese medical theory as the congenital foundation. The growing development of the body depends on kidney essence [33]. The “kidney is mainly for storage, and it is the place where essence exists,” according to *Elements: Six-section Theory of Viscera* [34]. The characteristic of the kidney is prone to storing essence, and the essence of the body should be stored instead of being depleted. The subjects of this study are male youths with drug addiction who indulge themselves in excessive sex after taking drugs, and most of them suffer from damaged kidney essence and yang-qi deficiency after long-term drug use, which results in renal yang deficiency. Most of the drugs are bitter and warm in nature, and they consume and damage the yang-qi of the spleen and stomach. This is why the syndrome of drug addiction is commonly associated with yang-qi deficiency.

According to TCM, narcotics belong to the material of warmth and dryness that reduces yin. Long-term and excessive drug use may lead to an unnoticeable consumption of yin fluid, resulting in loss and deficiency of yin fluid. When the body loses moisture and nourishment, it becomes yin deficient with noticeable symptoms. Benzedrine-based drugs are central nervous stimulants. The excessive intake of these drugs will lead to irreversible brain damage, and the patient will behave differently from ordinary people, with symptoms such as reduced sleep, excessive self-confidence, mania, delusions, and other abnormal mental symptoms. According to *Elements: the Theory of Linglan Secret Codes*, “the heart is the chief organ that governs the person and the place where your spirit forms.” [34] TCM deems that people's mind activities are closely related to the function of the heart. When the function is normal, the person is clear-minded, quick-thinking, and energetic. According to *Elements: Chapter on Formation of the Five Internal Organs* [34], blood is the basis of major matters of mental activity. When blood is full, the functional activities of a person are normal and the mental activity of the person is in a normal physiological state. Blood comes from yin-qi in the blood vessels, and the yin-qi originates from the essence of water and grain in the spleen and stomach. Excessive drug use results in loss of appetite, inadequate food intake, and spleen and stomach injury, which further leads to insufficient blood generation and blood deficiency.

In this study, there was no significant difference between the intervention group and the control group in the syndromes of yang deficiency, qi deficiency, or yang-qi deficiency before the intervention, which indicated baseline equilibrium comparability. After the intervention of the TCM-guided diet, all the *P* values obtained from the comparison between the two groups indicated the improvement of yang deficiency. In the pre-post comparison, the *P* value for qi deficiency of the people with drug addiction in the intervention group showed improvement although the syndromes of yang deficiency and yang-qi deficiency also showed improvement. In the control group, the syndromes of yang deficiency, qi deficiency, and yang-qi deficiency of drug addicts were all aggravated.

In summary, the analysis of the results showed that the yang-warming and qi-tonifying TCM-guided diet can improve the syndrome of qi deficiency in male youths with drug addiction, but the improvement in the accumulated syndrome elements of yang-qi deficiency was not so apparent. However, the TCM-guided diet could delay the deterioration associated with the syndrome of yang-qi deficiency. The reasons for the improvement may be summarized as follows: ① After using benzedrine-based drugs, an individual with drug addiction exhibits an elated state driven by adrenaline surges that are extremely qi consuming. After abstinence, the body loses the power of the drugs to support yang qi, and then the syndrome of yang-qi deficiency appears. The TCM-guided diet tonifies qi and gradually adds vitality to the body after abstinence. This might be the reason for the improvement in the syndrome of qi deficiency. ② According to TCM, it is better to reach a balance between yin and yang. Therefore, the fundamental goal in TCM is to adjust yin and yang, remedy defects, rectify errors, and rebalance yin and yang to make sure yin and yang are in equilibrium. Yin and yang are interdependent and mutually promoting. Those who are good at supplementing yang must seek yang in yin. When yang is assisted by yin, it can generate infinite benefits. It is difficult to achieve a balance between yin and yang when only warming yang and tonifying qi without supplementing yin fluid. That might be the reason the treatment effect obtained by only tonifying yang is not as good. ③ The food and drugs that warm yang and tonify qi are more or less warm and dry in nature. A long-term use of these kinds of foods and drugs may damage yin liquid and result in both yin deficiency and yang deficiency. The therapy that only tonifies yang-qi is therefore ineffective. ④ Addictive drugs or narcotics easily consume yang qi. Unfortunately, there are fewer products for strengthening and invigorating the spleen in the yang-warming and qi-tonifying medicinal diets. When the water and grain essence is not strong enough to generate vitality, the diet without strength to generate vitality could hardly reach efficacy in absorption. We may conclude that although the yang-warming and qi-tonifying medicinal diet could not completely improve the syndrome of yang-qi deficiency, it could delay the deterioration process associated with the syndrome of yang-qi deficiency in male youths with drug addictions. In the future, we can consider adjusting the prescription of the diet, adding some ingredients that are good for tonifying yin, and invigorating the spleen when appropriate. In this way, we can further explore the curative effect of a TCM-guided diet for people with drug addiction with yang-qi deficiency syndrome.

Before the intervention, there was no significant difference in the syndromes of yin deficiency, blood deficiency, or yin-blood deficiency between the intervention group and the control group, which indicated baseline equilibrium and comparability. After intervention with the TCM-guided diet, *P* values from the comparison of the syndrome improvements of the individuals with drug addiction between the two groups in yin deficiency, blood deficiency, and yin-blood deficiency indicated statistical significance. In regard to the pre- and post-therapy comparison, the syndromes of yin deficiency, blood deficiency, and yin-blood deficiency of the drug addicts in the intervention group improved, while those of yin deficiency, blood deficiency, and yin-blood deficiency in the control group all increased, but none of the differences were statistically significant.

The outcomes unveiled that the yin-tonifying and blood-nourishing TCM-guided diet could improve the syndromes of yin deficiency and blood deficiency in male youths with drug addiction in rehabilitation, and the curative effect was better than those associated with conventional diets. Long-term and excessive use of benzedrine-based drugs could lead to yin-blood deficiency. The yin-tonifying and blood-nourishing diet could moisten yin liquid and reduce the consumption of yin liquid caused by addictive drugs. According to TCM, the spleen and stomach are the source areas of qi and blood. When the spleen and stomach are damaged, the generation of qi and blood is insufficient and blood deficiency is difficult to replenish. Qi and blood are of the same origin. In the treatment of blood deficiency syndrome, both qi and blood should be supplemented at the same time because qi supplementation can facilitate the operation of blood. When treating those who are suffering from yin-blood deficiency, we can add some ingredients that will strengthen the spleen and stomach, tonify qi, and supplement blood when tonifying yin and nourishing blood to improve the functions of the spleen and stomach. In this way, we can make the generating sources, the spleen and stomach, more powerful, resulting in sufficient generation of blood. Only when blood deficiency is remedied can the syndrome of blood deficiency be improved. TCM basic theory insists that when supplementing yin, one must try to strengthen yin while consolidating yang. Only when yin is improved and yang is increased can the generation fountain never be exhausted. It is suggested that in future research, some yang-tonifying ingredients be added in the yin-tonifying and blood-nourishing diet prescription to promote the balance of yin and yang.

Based on TCM theory, persons with different body constitutions are required to eat different foods by taking into account their dietary habits. According to individual tastes, foods are cooked into different types, such as soups or congee [35]. In addition, the TCM-guided diet in this study included *Codonopsis* radix, *Astragalus* radix, *Angelicae sinensis* radix, *Dioscoreae* rhizoma, Polygonati odorati rhizoma, and Jujubae fructus. Their main ingredients or extracts have good effects of warming yang, replenishing qi, nourishing yin, and nourishing blood, which improve neurological function and outgrowth axonal, offer certain complementary cognitive benefits, enhance physical strength and the immune system, and stimulate haematopoiesis in the body [36–41]. This is the only study to verify that the yin-tonifying and blood-nourishing TCM-guided diet is effective for male youths with drug addiction with yin-blood deficiency. Other researchers need to extend the population of subjects for further exploration.

Our study had some limitations: the sample size was small, and it was not a multicenter and double-blind experiment. Therefore, the influence of psychological factors may exist in regard to the collection of accumulated TCM syndrome elements. At present, the understanding of TCM in regard to the mechanism of new addictive drugs has not been unified, and research is needed in the future to further study their aetiology and pathogenesis.

The innovation of this research lies in the TCM-guided diet intervention for male youths with yang-qi deficiency and yin-blood deficiency in the rehabilitation period. According to the principle of syndrome differentiation, Chinese medicine is used to increase the physical function of the compulsory isolation and detoxification of male youths during rehabilitation. The TCM-guided diet assists the youths in compulsory isolation with detoxification as a model of good public health value and social benefits.

In conclusion, through baseline comparison, the intervention group was worse, the control group was better, although there was no significant difference. After the TCM-guided diet intervention, the intervention group improved, but the control group unchanged or even worsened. The change between the two groups was statistically significant. Therefore, a TCM-guided diet can delay the worsening of yang-qi deficiency syndrome, improve yin-blood deficiency syndrome, and improve the prognosis of male youths undergoing drug detoxification during the rehabilitation period.

## Figures and Tables

**Table 1:** TCM-guided diet menu for male youth undergoing drug detoxification with yang-qi deficiency and yin-blood deficiency.

Warms yang and invigorates qi diet	Steamed duck of Sijun, Miqin of Huaishan. Solid lean broth, black rice dangshen porridge, red Qitian Qiji, Shenqi beef soup, fried walnut with leek, Huangqi yam porridge, dangshen cooked rabbit meat, Angelica ginger and mutton soup, Chinese yam porridge, Guiqi stewed chicken, Chinese yam eel soup, lotus seed stewed pork belly, millet jujube porridge, Chinese yam steamed carrot, crucian carp stewed tofu, lotus seed and lily yam porridge, Angelica Duzhong mutton soup, steamed chicken, Astragalus steamed carp, Astragalus steamed carp and carp fish soup, reinforcing deficiency and reinforcing qi congee

Nourishing yin and blood diet	*Salvia miltiorrhiza* Yuzhu stewed duck, lotus lily lean broth, black rice red jujube congee, steamed chicken with yam, medlar clam soup, Shengui pig liver soup, medlar congee, lotus seed stewed duck, peanut rice jujube stewed porridge, mung bean lily congee, raw ground yellow chicken, pig heart jujube kernel soup, spinach pig liver soup, small rice jujube congee, dangshen wolfberry stewed white duck, Yuzhuxinzi, lotus seed lily yam porridge, mung bean kelp duck soup, yellow essence roasted chicken, red wolfberry roasted live fish, ginseng Tremella porridge

**Table 2:** Diagnostic criteria of syndrome elements.

Diagnostic criteria of disease location syndrome elements	Diagnostic criteria of disease venereal syndrome elements	Significance
Integral of disease location syndrome elements ≥100	Integral of disease venereal syndrome elements ≥100	The severity of symptoms is moderate
		Quantitative diagnostic value multiplied by 1.5 for severe symptoms
		Quantitative diagnostic value multiplied by 0.7 for mild symptoms
Grade	Contribution integral of differentiation elements	
Level 0	Integral < 70	No pathological changes
Level 1	70 ≤ Integral < 100	Mild pathological changes
Level 2	100 ≤ Integral < 150	Moderate pathological changes
Level 3	Integral ≥ 150	Severe pathological changes

**Table 3:** Demographic characteristics of the two groups at baseline.

Index	Intervention group (*n* = 62)	Control group (*n* = 61)	*t*/*χ*^2^ value	*P* value
*Demographic characteristics*				
Age	22.10 ± 2.63	21.88 ± 2.90	0.442	0.660
*Ethnicity*			1.068	0.301
Han	61 (98.4)	58 (95.1)		
Ethnic minority	1 (1.6)	3 (4.9)		
*Occupation*			1.432	0.698
Worker	6 (9.7)	3 (4.9)		
Farmer	2 (3.2)	2 (3.3)		
Self-employed	9 (14.5)	12 (19.7)		
Other	45 (72.6)	44 (72.1)		
*Marital status*			0.359	0.549
Unmarried	61 (98.4)	59 (96.7)		
Married	1 (1.6)	2 (3.3)		
*Education level*			2.877	0.237
Elementary school	21 (33.9)	14 (23.0)		
Secondary school	35 (56.5)	36 (59.0)		
Junior high school or secondary specialized school and above	6 (9.7)	11 (18.0)		
*Family economic situation*			0.002	0.999
Low (<10000 RMB)	11 (17.7)	11 (18.0)		
Medium (10000∼30000 RMB)	48 (77.4)	47 (77.0)		
High (>30000 RMB)	3 (4.8)	3 (4.9)		
*Medical insurance form*			4.615	0.329
Self-pay	34 (54.8)	28 (45.9)		
Medical insurance for urban workers	20 (32.3)	27 (44.3)		
Rural cooperative medical service	1 (1.6)	3 (4.9)		
Commercial medical insurance	1 (1.6)	1 (1.6)		
Missing	6 (9.7)	2 (3.3)		

**Table 4:** Drug abuse characteristics of the two groups at baseline.

Index	Intervention group (*n* = 62)	Control group (*n* = 61)	*χ* ^2^ value	*P* value
*Years of drug abuse*			1.256	0.869
1 year	2 (3.2)	3 (4.9)		
2 years	7 (11.3)	8 (13.1)		
3 years	18 (29.0)	20 (32.8)		
4–6 years	28 (45.2)	26 (42.6)		
7–15 years	7 (11.3)	4 (6.6)		

*Drug types (multiple selection)*			23.741	0.254
Methamphetamine	62	60		
Ecstasy	16	7		
Ketamine (K powder)	37	36		
Heroin (yellow peel/white powder)	1	2		
Opium	1	0		
Cocaine	0	1		
Marijuana	12	11		
Ephedrine	4	4		
Pethidine	0	1		
Morphine	1	0		
Methadone	1	0		
Somedon	1	0		
Tramadol	0	1		
GHB	0	2		
Other	0	1		

*Drug abuse patterns (multiple selection)*			31.611	0.386
Smoke gun suction	42	37		
Ironing suction	24	32		
Nasal suction	19	23		
Pipe suction	20	26		
Cigarette smoking	5	8		
Oral administration	3	6		
Dissolve in beverage	3	6		

*Daily amount of drug abuse*			1.859	0.602
0.1–0.5 g	21 (33.9)	20 (32.8)		
0.5–1.0 g	26 (41.9)	32 (52.5)		
1.0–2.0 g	10 (16.1)	8 (13.1)		
2 g or more	3 (4.8)	1 (1.6)		

*Frequency of drug detoxification*			6.801	0.079
1 time	57 (91.9)	56 (91.8)		
2 times	0 (0.0)	4 (6.6)		
3 times	4 (6.5)	1 (1.6)		
4 times	1 (1.6)	0 (0.0)		

**Table 5:** Score changes in yang deficiency syndrome elements in the two groups.

Indicators	Intervention group (*n* = 62)	Control group (*n* = 61)	*t* value^*∗*^	*P* value
Baseline value	251.37 ± 112.39	224.52 ± 112.81	1.322	0.189
Postintervention	239.71 ± 121.46	281.23 ± 149.28	−1.693	0.093
Postintervention difference (post-pre)	−13.95 (−76.95, 52.36)	40.70 (−22.80, 139.75)	−3.059^*∗*^	0.003
Improvement (%)	4.63	−25.26		
Intragroup *t* (P)	0.923 (0.359)	−3.075 (0.003)		

Intragroup *t* (P) represents the statistics and *P* value of pairing *t*-test compared with that before treatment. ^*∗*^Mann–Whitney *U* test was adopted.

**Table 6:** Changes in qi deficiency syndrome element scores in the two groups.

Indicators	Intervention group (*n* = 62)	Control group (*n* = 61)	*t* value^*∗*^	*P* value
Baseline value	264.41 ± 106.65	227.77 ± 99.88	1.966	0.052
Postintervention	235.29 ± 115.47	264.68 ± 137.56	−1.284	0.202
Postintervention difference (post-pre)	−28.85 (−77.30, 37.50)	20.90 (−29.90, 108.15)	−3.303^*∗*^	0.003
Improvement (%)	11.01	−16.20		
Intragroup *t* (P)	2.690 (0.009)	−2.196 (0.032)		

Intragroup *t* (P) represents the statistics and *P* value of paired *t*-test compared with that before treatment. ^*∗*^Mann–Whitney *U* test is adopted.

**Table 7:** Change in the sum of scores of yang-qi deficiency syndrome elements in the two groups.

Indicators	Intervention group (*n* = 62)	Control group (*n* = 61)	*t* value^*∗*^	*P* value
Baseline value	515.78 ± 215.61	452.29 ± 210.08	1.654	0.101
Postintervention	475.00 ± 233.86	545.91 ± 284.95	−1.510	0.134
Postintervention difference (post-pre)	−38.05 (−131.48, 82.13)	67.80 (−47.85, 244.90)	−3.225^*∗*^	0.003
Improvement (%)	7.91	−20.70		
Intragroup *t* (P)	1.785 (0.079)	−2.686 (0.009)		

Intragroup *t* (P) represents the statistics and *P* value of paired *t*-test compared with that before treatment. ^*∗*^Mann–Whitney *U* test is adopted.

**Table 8:** Score changes in yin deficiency syndrome elements in the two groups.

Indicators	Intervention group (*n* = 62)	Control group (*n* = 61)	*t* value^*∗*^	*P* value
Baseline value	239.92 ± 98.19	208.66 ± 88.60	1.852	0.066
Postintervention	201.57 ± 106.39	229.82 ± 122.63	−1.365	0.175
Postintervention difference (post-pre)	−36.85 (−82.70, 15.38)	12.00 (−35.45, 80.15)	−3.538^*∗*^	0.001
Improvement (%)	15.98	−10.14		
Intragroup *t* (P)	4.034 (0.001)	−1.525 (0.133)		

Intragroup *t* (P) represents the statistics and *P* value of paired *t*-test compared with that before treatment. ^*∗*^Mann–Whitney *U* test was adopted.

**Table 9:** Score changes in blood deficiency syndrome elements in the two groups.

Indicators	Intervention group (*n* = 62)	Control group (*n* = 61)	*t* value^*∗*^	*P* value
Baseline value	233.19 ± 99.61	207.82 ± 85.15	1.517	0.132
Postintervention	205.98 ± 106.20	233.96 ± 119.73	−1.372	0.173
Postintervention difference (post-pre)	−22.40 (−72.23, 13.53)	20.00 (−45.95, 79.50)	−3.173^*∗*^	0.005
Improvement (%)	11.67	−12.58		
Intragroup *t* (P)	2.888 (0.005)	−1.877 (0.065)		

Intragroup *t* (P) represents the statistics and *P* value of paired *t*-test compared with that before treatment. ^*∗*^Mann–Whitney *U* test was adopted.

**Table 10:** Change in the sum of scores of yin-blood deficiency syndrome elements in the two groups.

Indicators	Intervention group (*n* = 62)	Control group (*n* = 61)	*t* value^*∗*^	*P* value
Baseline value	473.11 ± 194.16	416.49 ± 170.29	1.718	0.088
Postintervention	407.55 ± 209.53	463.78 ± 240.57	−1.383	0.169
Postintervention difference (post-pre)	−57.20 (−155.20, 21.93)	23.50 (−76.40, 157.30)	−3.419^*∗*^	0.002
Improvement (%)	13.86	−11.35		
Intragroup *t* (P)	3.579 (0.001)	−1.723 (0.090)		

Intragroup *t* (P) represents the statistics and *P* value of paired *t*-test compared with that before treatment. ^*∗*^Mann–Whitney *U* test was adopted.

**Table 11:** The vital signs before and after the intervention in the two groups.

Index	Baseline			After 3 months		
	Intervention group (*n* = 62)	Control group (*n* = 61)	*t* value (*P* value)	Intervention group (*n* = 62)	Control group (*n* = 61)	*t* value (*P* value)
Height (cm)	169.80 ± 5.95	168.87 ± 6.39	0.646 (0.520)	169.72 ± 5.97	168.81 ± 6.42	0.633 (0.529)
Weight (kg)	70.26 ± 11.02	68.28 ± 8.68	0.861 (0.392)	72.16 ± 11.73	69.14 ± 10.53	1.169 (0.246)
Systolic pressure (mmHg)	124.92 ± 11.20	128.61 ± 13.26	−1.289 (0.201)	124.94 ± 12.82	122.40 ± 12.04	0.882 (0.381)
Diastolic pressure (mmHg)	76.08 ± 10.30	74.97 ± 8.25	0.513 (0.610)	76.81 ± 9.41	74.55 ± 11.57	0.916 (0.363)
Pulse (times/points)	70 ± 11	68 ± 10	0.741 (0.461)	73 ± 14	73 ± 10	0.020 (0.984)

Intragroup *t* (P) represents the statistics and *P* value of the paired *t*-test compared with that before treatment. ^*∗*^Mann–Whitney *U* test was adopted.

## Data Availability

The data used to support the findings of this study are available from the corresponding author upon request.
